# Item

**Published:** 1899-01

**Authors:** 


### ITEM.

MODERN VACCINE PLANT.—The spirit of improvement has seized upon vaccine material and vaccination, and the result is glycerinated vaccine lymph, more successful vaccinations and much less suffering to the child. This new form is vaccine virus purified and preserved by means of glycerin and is now prepared in this country in the special vaccine laboratory of H. K. Mulford Company, recently erected on their biologic farm, at Glenolden, Delaware County, Pa. The laboratory is distinct from the general laboratory and is situated between the stables to which it is connected by venandas. This enables the employment of one while the other is being disinfected. The entire plant combines the best features of the most modern and improved vaccine establishments of Europe and is said to be second to none in the world.

